# Association of iKIR-mismatch model and donor aKIRs with better outcome in haploidentical hematopoietic stem cell transplantation for acute myeloid leukemia

**DOI:** 10.3389/fimmu.2022.1091188

**Published:** 2023-01-24

**Authors:** Yu Zhang, Chenjing Ye, Haojie Zhu, Youran Zhuang, Shaozhen Chen, Yingxi Weng, Jinhua Ren, Xiaofeng Luo, Jing Zheng, Xiaoyun Zheng, Jing Li, Lingqiong Lan, Yongxin Xie, Zhongchao Han, Jianda Hu, Ting Yang

**Affiliations:** ^1^ Fujian Medical University, Fuzhou, Fujian, China; ^2^ Department of Hematology, Fujian Medical University Union Hospital, Fujian Institute of Hematology, Fujian Provincial Key Laboratory of Hematology Fuzhou, Fujian, China; ^3^ School of Public Health, Fujian Medical University, Fuzhou, Fujian, China; ^4^ School of Medical Sciences, Faculty of Medicine & Health, University of Sydney, Sydney, Australia; ^5^ Chinese Academy of Medical Sciences, State Key Laboratory Experimental Hematology, Tianjin, China

**Keywords:** killer cell immunoglobulin like receptor, KIR ligand mismatch, KIR receptor-ligand mismatch, haploidentical hematopoietic stem cell transplantation, acute myeloid leukemia, natural killer cell

## Abstract

**Objectives:**

Killer cell immunoglobulin like receptor (KIR) can trigger the alloreactivity of NK cells. However, there is no clear consensus as to their function. Here, we investigated the potential influence of KIR mismatch and KIR alleles on the outcome of haploidentical hematopoietic stem cell transplantation (haplo-HSCT) in acute myeloid leukemia (AML) patients.

**Method:**

Data from 79 AML patients treated with haplo-HSCT were retrospectively analyzed. HLA-C genotyping was determined by the PCR-rSSO method. KIR, HLA-A and HLA-B genotyping was performed by the PCR-SSP method. Cox proportional hazards model and Kaplan-Meier survival curves were used for analysis.

**Results:**

Both KIR ligand mismatch (KLM) group and KIR receptor-ligand mismatch (RLM) group were associated with a decreased risk in aGVHD and relapse rate (RR), and better overall survival (OS) compared to the KIR ligand matching and receptor-ligand matching groups, respectively (aGVHD: KLM: p=0.047, HR:0.235; RLM: p<0.001, HR:0.129; RR: KLM: p=0.049, HR:0.686, RLM: p=0.017, HR:0.200;OS:KLM: p=0.012, HR: 0.298, RLM: p=0.021, HR:0.301). RLM was more accurate at predicting relapse and aGVHD compared with KLM (aGVHD: p=0.009; RR: p=0.039). Patients with greater number of donor activating KIRs (aKIR) had a lower incidence of aGVHD and relapse, and the benefits correlated with the increase in the number of donor aKIRs (aGVHD: p=0.019, HR:0.156; RR: p=0.037, HR:0.211). Patients with RLM and the highest number of donor aKIRs had the lowest RR, lowest incidence of aGVHD and best OS.

**Conclusions:**

Both KLM and RLM reduced the risk of aGVHD and relapse after haplo-HSCT in AML patients, and RLM showed superiority in predicting HSCT outcome. The synergistic effects of RLM and donor aKIRs can provide a better donor selection strategy to improve haplo-HSCT outcome in AML patients.

## Introduction

Allogeneic hematopoietic stem cell transplantation (allo-HSCT) offers hope for a cure to a growing number of patients with acute myeloid leukemia (AML). In clinical practice, the most common criteria for donor selection is based on HLA-matching, which can significantly reduce the incidence of graft-versus-host disease (GVHD). However, HLA-matching may result in higher relapse rate (RR), which is responsible for 33% of deaths in unrelated donor (URD) HSCT and 47% of deaths in matched sibling donor (MSD) HSCT beyond 100 days post-transplant according to the data from Center for International Blood and Marrow Transplant Research (CIBMTR) (1, 2). Thus, a new donor selection strategy in addition to HLA has been an intense field of research.

NK cells were believed to have an anti-leukemia effect, while simultaneously providing GVHD protection. Their surface receptors, killer cell immunoglobulin-like receptors (KIRs), play a critical role in NK alloreactivity through interaction with their ligands, which include human leukocyte antigen-C (HLA-C), HLA-E, HLA-BW4, HLA-A, etc. to transmit inhibitory or activating signals (3). KIR genes are located on chromosome 19q13.4 (4). 16 KIR genes (including two pseudogenes) have been identified. The name of the gene is based on its structure, including the number of Ig-like domains (D) and the length of the tail (S or L). Six genes with short tails (KIR2DS or KIR3DS) are activating KIR genes that encode activating receptors, while the eight genes with long tails (KIR2DL or KIR3DL) are inhibitory KIR genes encoding inhibitory receptors (but except KIR2DL4). According to the activating genes, KIRs can be further divided into haplotype A and B. Haplotype A has only one activating gene (KIR2DS4) while haplotype B has five activating KIR genes (KIR2DS1, 2, 3, 5, and 3DS1) (5, 6). Inhibitory KIRs (iKIRs) establish tolerance by transmitting inhibitory signals after ligand binding. After HSCT, when the donor NK cells express iKIR that fails to bind to its ligand on the recipient target cells, based on the “missing self” recognition theory, the alloreactivity of NK cells is triggered, resulting in NK cell-mediated lysis (7, 8). Activating KIRs (aKIRs) transmit activating signals that induce NK activation and cellular cytotoxicity when binding to their ligand; thus, aKIRs play an important role in regulating NK cell activity and influencing the outcome of HSCT (9). As KIR and HLA genes are located on different chromosomes and independently inherited, KIRs can provide an additional donor selection strategy besides HLA (6). In addition, KIR genotyping has the advantages of being simple, economic and informative, and donor selection based on KIR matching and KIR locus provides a convenient and effective way to improve the prognosis of HSCT.

**Figure 1:**
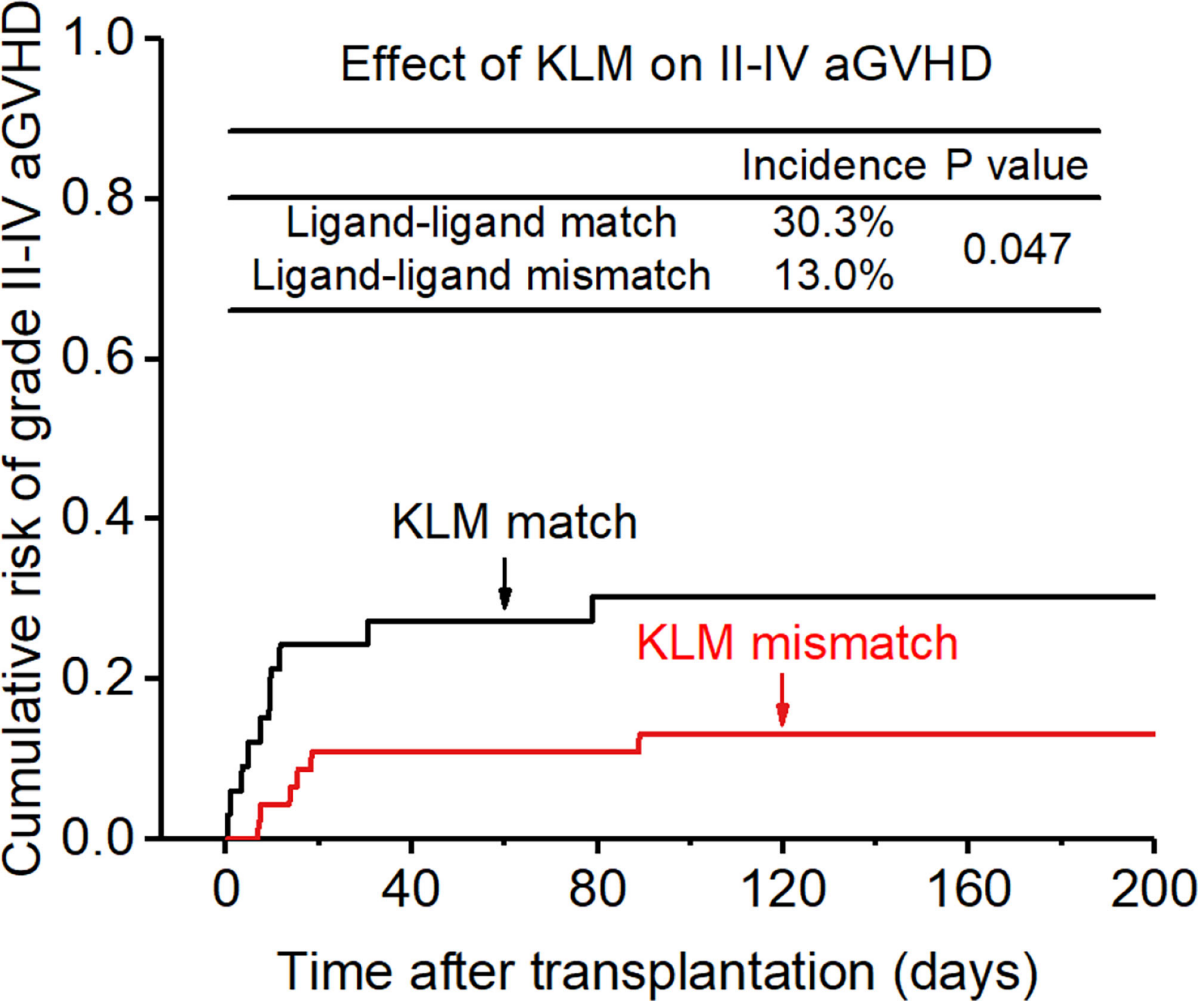
Effect of killer cell immunoglobulin-like receptor (KIR) ligand-ligand mismatch (KLM) on grade II-IV acute-graft-versus-host disease (aGVHD).

While numerous retrospective studies have indicated that donor selection based on KIRs may improve transplantation outcomes, there are still significant controversies about KIRs and their role in HSCT outcome (10). Conflicting findings can be concluded to few commonalities in treatment, patient cohorts and study protocols between different transplantation centers. Furthermore, there is still no consensus on the models for assessing KIR compatibility between recipients and donors. There are currently three models that have been accepted. Derived from the hypothesis of ‘missing-self’ (7), Ruggeri et al. established ligand-ligand mismatch model in 2002, which mainly focus on the incompatibility between the donor KIR ligand and recipient KIR ligand (11). Later, Leung et al. proposed receptor-ligand mismatch model in 2004, which was defined by the incompatibility between the donor KIR and recipient KIR ligand (10). Gagne et al. raised model of receptor-receptor mismatch in 2002, taking the incompatibility as a mismatch between the donor KIR and recipient KIR (12). Because KIR mismatch in unrelated HSCT were closely related to the presence of B-haplotype activating genes (KIR2DS3 and KIR2DS5) in donor, McQueen et al. reclassified receptor-receptor mismatch model as the one considering the mismatch between donor and recipient for the B KIR haplotypes (13). (Details of three mismatch models were presented in **Supplementary Table 1** and **Figure 1**). However, few analyses have compared the clinical relevance between these models, especially for the very similar KIR receptor-ligand mismatch (RLM) and KIR ligand-ligand mismatch (KLM) models. Taken together, KLM can be defined as a missing KIR ligand in a recipient for a KIR ligand that is present in donor (i.e., ligand-ligand mismatch if the donor has a ligand that is absent in the recipient). RLM can be defined as a missing KIR ligand in a recipient for a KIR inhibitory receptor that is present in donor (i.e., receptor-ligand mismatch if the donor has an inhibitory receptor for which the cognate ligand is absent in the recipient) (10, 11). Consequently, more research is required to ascertain which model is the most beneficial to patient care (10). Besides, studies focusing on the effect of each KIR genotype separately indicate that some KIR genotypes, especially some aKIRs, such as 3DS1 or 2DS1, and KIR haplotype B, may contribute to better survival in AML patients (14–16). Nevertheless, others have shown that the function of some KIR genotypes is not entirely clear, and some studies suggest that they can aggravate the severity of GVHD and worsen the prognosis (15–17). Thus, the role of KIRs on HSCT outcome remains ill-defined at the present time.

In this study, we retrospectively analyzed the charts from 79 AML patirents who underwent haplo-HSCT to determine the potential influence of different KIR mismatch models and genotypes in order to provide an optimal strategy for donor selection based on KIRs.

## Patients and methods

### Patients

Seventy-nine patients diagnosed with AML who consecutively underwent haploidentical-HSCT between May 2015 and May 2020 in the department of hematology of the Fujian Medical University Union Hospital were selected. AML was diagnosed based on the National Comprehensive Cancer Network practice guidelines. This retrospective chart review study was approved by the Human Investigation Committee of the Fujian Medical University Union Hospital, and was conducted in accordance with the ethical standards of the institutional and national research committee and with the Helsinki Declaration. Patients with no DNA available for KIR typing were excluded from the study. Patients lost to follow-up were also excluded.

### KIR and HLA genotyping

Blood samples were collected from the patients and their donors for HLA typing (18). Genomic DNA was extracted from each sample with a commercial kit (Qiagen, Hilden, Germany) according to the manufacturer’s instructions. HLA-A and -B alleles were genotyped by polymerase chain reaction using sequence-specific primers (PCR-SSP) (TBG, Taipei, Taiwan). HLA-C genotyping was assessed by the reverse hybridization line probe assay with sequence-specific oligonucleotides probes (rSSO) using INNO-LiPA^®^ HLA-C kit (Fujirebio Europe, Technologiepark 6, B-9052 Gent, Belgium) according to the rmanufacturer’s instructions. HLA alleles were then assigned using software programs in each kit.

KIR genotyping was performed by the PCR-SSO method using a commercially available kit (Gen-Probe Lifecodes KIR-SSO typing kit; Immucor Transplant Diagnostics, Inc, Stamford, USA) containing 20 different oligonucleotide probes for known KIR genes or alleles (KIR2DL1, KIR2DL2*001-3/5, KIR2DL2*004, KIR2DL3, KIR2DL5, KIR3DL1, KIR2DS1, KIR2DS2, KIR2DS3, KIR2DS4*whole exon 4, KIR2DS4*whole exon 5, KIR2DS4*deleted exon 5, KIR2DS5, KIR3DS1, KIR3DS1*049N). The amplicons were quantified on the Luminex 200 flow analyzer (Luminex Corporation, Austin, TX, USA) and analyzed using the Quick-Type for Lifecodes software (version 3.3, Immucor Transplant Diagnostics, Inc, Stamford, CT, USA) to generate the KIR data (19).

### Transplant protocol

Patients were treated with the FA5-BUCY regimen consisting of Fludarabine (30 mg/m^2^/day) and high-dose Cytarabine (Ara-C; 2 g/m^2^/day) for 5 consecutive days from day -13 to day -9, followed after 1 day of rest by busulfan (BU; 3.2mg/kg/day) from day -7 to day -5 and cyclophosphamide (CY; 1.8g/m^2^/day) from day -4 to day -3 (17).

Out of the 79 patients, only one patient underwent bone marrow (BM) transplantation, while all others received peripheral blood (PB) stem cell transplantation. Peripheral blood stem cells were obtained after mobilization with granulocyte colony-stimulating factor (G-CSF; 5 μg/kg of body weight per day for 5 days), as described by Huang et al. (20–22) The target total mononuclear cell counts from BM and PB were >4x10^8^/kg of recipient weight. All patients received fresh grafts containing a median of 7.9x10^8^ mononuclear cells/kg (range 4.2-21.4x10^8^/kg) and 5.3x10^6^ CD34+ cells/kg (range 2.6–28x10^6^/kg) on day 0.

GVHD prophylaxis consisted of rabbit anti-thymocyte globulin (ATG, Thymoglobulin, Genzyme, 10mg/kg), or ATG-Fresenius^®^ (40mg/kg)) from day -4 to day -1, cyclosporine A (plasma level 100–250 ng/ml, starting from day −10, and tapered from the second or third month post-transplant, if no signs of GVHD), mycophenolate mofetil (5 mg/kg BID, starting from day +7, tapered after engraftment), and short-term methotrexate (MTX; 15 mg/m^2^ on day +1, and 10 mg/m^2^ on day +3 and +6) (19).

### Definition

The primary end points were overall survival (OS, defined as the time to death from any cause), occurrence of aGVHD (defined according to established criteria), and relapse rate (RR); relapsed AML was defined as >5% blasts in BM aspirates in patients who achieved complete cytological remission after the first or second induction treatment. Non-relapse mortality (NRM) was defined as death from any cause other than relapse. Patient disease risk index was classified as favorable/intermediate or unfavorable based on cytogenetic abnormalities (favorable: t(8;21), inv.(16) and t(15;17); intermediate: normal cytogenetics; unfavorable: all other chromosomal abnormalities).

### Statistical analysis

All data were analyzed using SPSS 22.0 (IBM, Armonk, NY, USA) and R project 3.6.1 software (http://www.r-project.org). OS was calculated using the Kaplan–Meier method and compared using the log-rank test. The cumulative incidences of aGVHD, cGVHD and relapse were estimated *via* the competing-risks model and compared using the Gray test. All variables with a p-value of <0.10 in the univariate analysis were then included in the multivariate analysis. For multivariate analyses, the Cox proportional hazards model was applied, using a forward stepwise approach. A p value of < 0.05 was considered as significant.

## Results

### Characteristics of patients and donors

Demographic data of the population studied are presented in **Table 1**. Among the 79 AML patients receiving haplo-HSCT at our center, 49 were male (62.0%) and 30 were female (38.0%), with a median age of 25 years (1-68 years). The median mononuclear cell (MNC) and CD34+ cell counts in the grafts were 7.9x10^8^/kg (range 4.2-21.4x10^8^/kg) and 5.3x10^6^/kg (range 2.6–28x10^6^/kg), respectively. All patients received a myeloablative conditioning (MAC) regimen. ATG-Fresenius was used in 33 patients (41.8%) while the other 46 (58.2%) received ATG as part of their conditioning regimen. Forty-nine patients (62.0%) were at CR1 at the time of transplantation, and 30 (38.0%) were not. Based on cytogenetic abnormalities, 26 (32.9%), 28 (35.4%) and 25 (31.6%) patients had favorable, intermediate or unfavorable disease risk index, respectively. The median follow-up was 24 months.

**Table 1:** Clinical features from patients and donors.

Variables	All patients	KIR ligand-ligand mismatch			KIR receptor-ligand mismatch		
	(N=79)	Match (N=33)	Mismatch (N=46)	*p*	Match (N=18)	Mismatch (N=61)	*p*
Median patient age (years)	25	24	26	0.983	25	24	0.901
Median donor age (years)	34.5	36	31.5	0.123	33	35	0.889
Median MNC (X10^8^/Kg)	7.9	7.9	8.1	0.824	8.3	7.7	0.307
Patient sex				0.100			0.107
Male	49 (62.0%)	19 (57.6%)	30 (65.2%)		10 (55.6%)	39 (63.9%)	
Female	30 (38.0%)	14 (42.4%)	16 (34.8%)		8 (44.4%)	22 (16.1%)	
Donor sex				0.414			0.676
Male	58 (73.4%)	25 (75.8%)	33 (71.7%)		14 (77.8%)	44 (72.1%)	
Female	21 (26.6%)	8 (24.2%)	13 (28.3%)		4 (22.2%)	17 (27.9%)	
Blood-type mismatch				0.621			0.823
Identical	37 (46.8%)	16 (48.4%)	21 (45.7%)		8 (44.4%)	29 (47.5%)	
Mismatch	42 (53.2%)	17 (51.5%)	25 (54.3%)		10 (55.6%)	32 (52.5%)	
Disease status				0.605			0.557
CR1	49 (62.0%)	21 (63.6%)	28 (60.9%)		12 (66.7%)	37 (60.7%)	
> CR1	30 (38.0%)	12 (36.4%)	18 (39.1%)		6 (33.3%)	24 (39.3%)	
Disease risk index				0.127			0.496
Favorable	26 (32.9%)	10 (30.3%)	16 (34.8%)		6 (33.3%)	20 (32.8%)	
Intermediate	28 (35.4%)	12 (36.4%)	16 (34.8%)		6 (33.3%)	22 (36.1%)	
Unfavorable	25 (31.6%)	11 (33.3%)	14 (34.0%)		6 (33.3%)	19 (31.1%)	
ATG or ATG-F				0.792			0.289
ATG-f	33 (41.8%)	14 (42.4%)	19 (41.3%)		7 (38.9%)	26 (42.6%)	
ATG	46 (58.2%)	19 (57.6%)	27 (58.7%)		11 (61.1%)	35 (57.4%)	

P-value from Fisher’s exact or chi-square tests. Data presented are n (%) unless otherwise indicated. *Disease risk index was defined by genetic abnormality of cytogenetic data available for 79 patients: numbers and percentages of patients are given; Favorable: t (8;21) (q22;q22.1), RUNX1-RUNXQT1; inv (16) (P13.1q22) or t (16;16) (p13,1;q22); CEBF-MYH11; Biallelic mutated CEBPA; Mutated NPM1 without FLT3-ITD or with FLT3-ITD low; Intermediate: Mutated NPM1 and FLT3-ITD high; Wild-type NPM1 without FLT3-ITD or with FLT3-ITD low; t (9;11) (p21.3;q23.3); MLLT3-KMT2A; Cytogenetic abnormalities not classified as favorable or unfavorable; Unfavorable: t (6;9) (p23;q34.1); DEK-NUP214;t (v;11q23.3);KMT2A rearranged; t (9;22) (q34.1;q11.2); BCR-ABL1;inv (3) (q21.3q26.2) or t (3;3) (q21.3;q26.2); GATA2;MECOMO (EVI1); -5 or del (5q);-7;-17/abn (17p); complex karyotype; monosomal karyotype; Wild-type NPM1 and FLT3-ITD high; Mutated RUNX1; Mutated ASXL1;Mutated TP53.

### aGVHD and cGVHD

Following transplantation, 16 patients (20.3%) developed grade II–IV aGVHD and 7 (8.7%) developed grade III–IV aGVHD (severe aGVHD).

As the heterogeneity of the patient cohort may affect the results, several other factors that may influence the severity of GVHD were taken into consideration, including donor-recipient HLA matching, gender of donor, age of recipient, patients prophylactically treated with ATG or ATG-F and donor type (related or unrelated). After adjusting for these related factors, both univariate and multivariate analyses showed that donor-recipient KLM, RLM and the number of donor aKIRs were closely associated with a decreased risk in aGVHD (**Table 2**).

**Table 2:** Multivariate analysis of factors associated with transplant outcome.

Outcome and significant factors	*p* value	HR (95% CI)
1. II-IV aGVHD		
Ligand-ligand match vs mismatch	0.047	0.235 (0.060-0.913)
Receptor-ligand match vs mismatch	<0.001	0.129 (0.046-0.361)
Number of donor aKIR <3 vs ≥3	0.019	0.156 (0.462-0.932)
2. III-IV aGVHD		
Ligand-ligand match vs mismatch	0.049	0.373 (0.135-0.972)
Receptor-ligand match vs mismatch	0.021	0.171 (0.038-0.770)
Number of donor aKIR <3 vs ≥3	0.024	0.165 (0.035-0.786)
3. Relapse rate		
Ligand-ligand match vs mismatch	0.049	0.686 (0.488-0.964)
Receptor-ligand match vs mismatch	0.017	0.200 (0.054-0.747)
Number of donor aKIR <3 vs ≥3	0.037	0.211 (0.049-0.911)
Disease risk index Favorable, Intermediate vs Unfavorable	0.007	3.568 (1.195-10.655)
Disease status CR1 vs >CR1	0.018	2.201 (1.053-2.759)
Donor haplotype A/A vs B/X	0.023	0.174 (0.049-0.619)
4. Overall survival		
Disease status CR1 vs >CR1	0.007	2.654 (1.481-3.890)
Disease risk index Favorable, Intermediate vs Unfavorable	0.006	4.132 (1.231-14.365)
Ligand-ligand match vs mismatch	0.012	0.298 (0.116-0.769)
Receptor-ligand match vs mismatch	0.021	0.301 (0.108-0.894)
Donor haplotype A/A vs B/X	0.029	0.293 (0.122-0.703)

Significant factors with p < 0.05 are in bold type.

**Figure 2:**
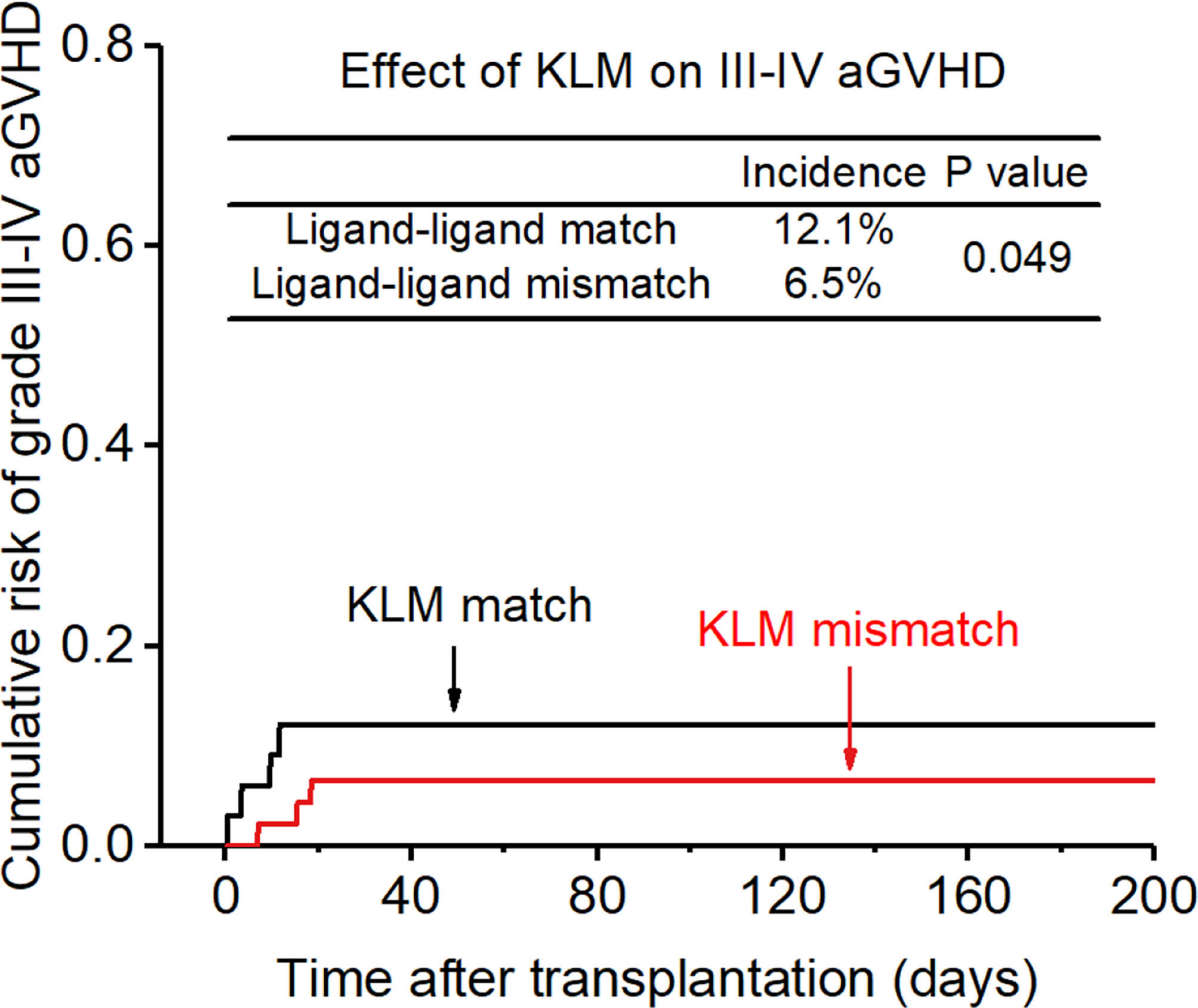
Effect of killer cell immunoglobulin-like receptor (KIR) ligand-ligand mismatch (KLM) on grade III-IV acute-graft-versus-host disease (aGVHD).

**Figure 3:**
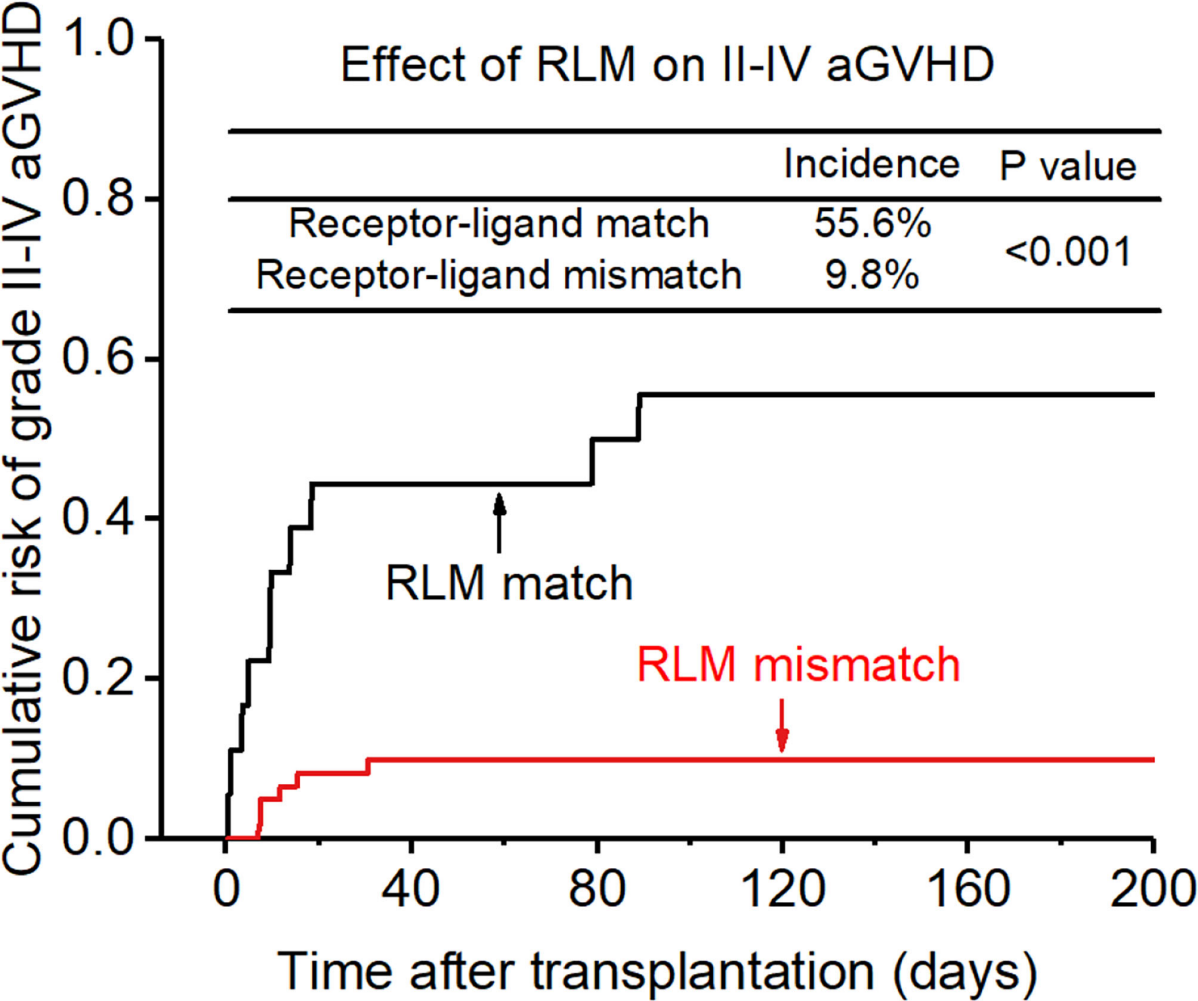
Effect of killer cell immunoglobulin-like receptor (KIR) receptor-ligand mismatch (RLM) on grade II-IV acute-graft-versus-host disease (GVHD).

**Figure 4:**
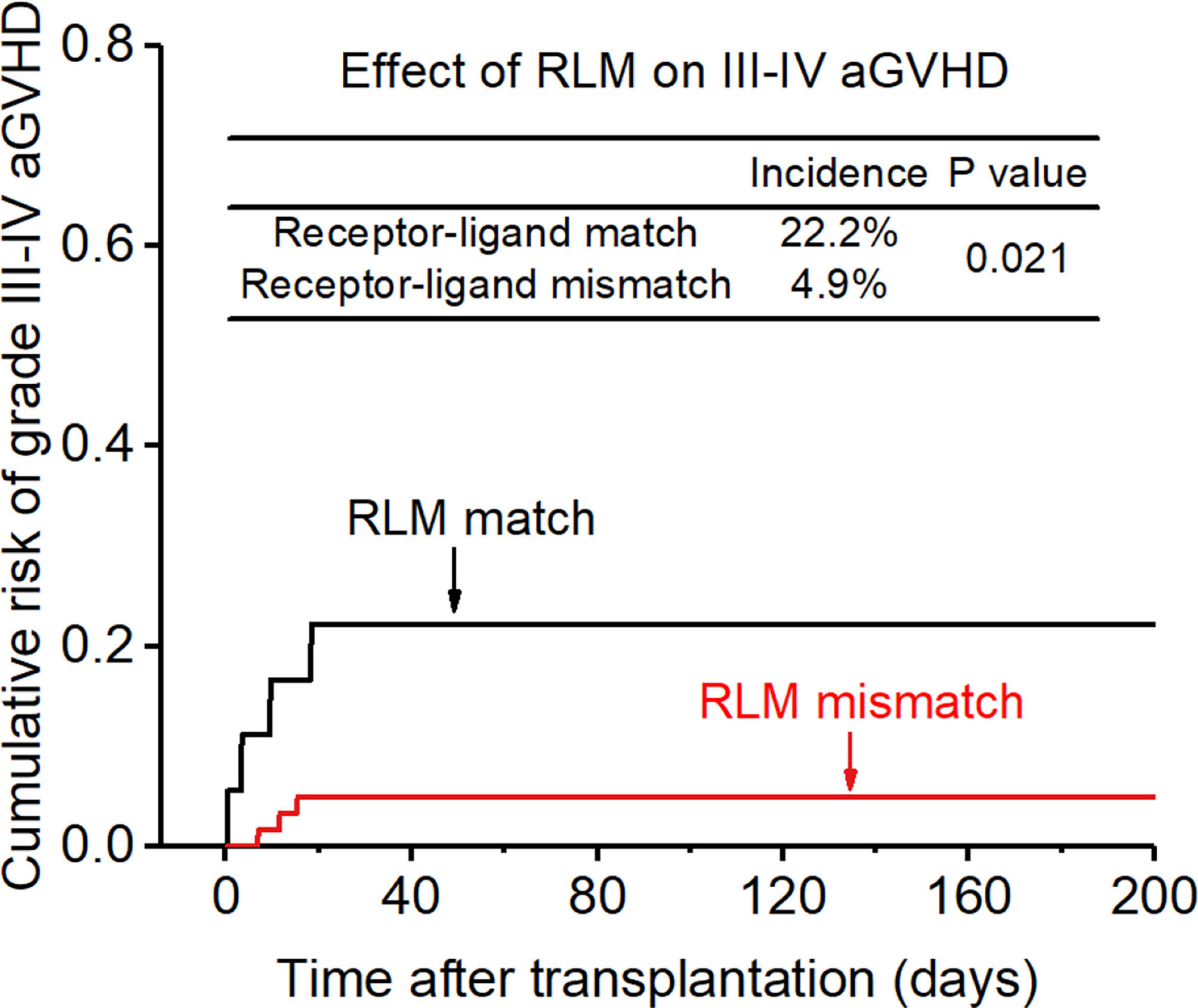
Effect of killer cell immunoglobulin-like receptor (KIR) receptor -ligand mismatch (RLM) on grade III-IV acute-graft-versus-host disease (aGVHD).

The incidence of grade II-IV aGVHD and III-IV aGVHD in the KLM group was significantly decreased compared to that in the matched group (II-IV: 13.0% versus 30.3%, p=0.047, HR=0.235; III-IV: 6.5% versus 12.1%, p=0.049, HR=0.373; **Figures 1**, **2**). In addition, the RLM group also showed a significant aGVHD reduction effect in both grade II-IV and III-IV aGVHD (II-IV: 9.8% versus 55.6%, p<0.001, HR=0.129; III-IV: 4.9% versus 22.2%, p=0.021, HR=0.171; **Figures 3**, **4**). The receptor-ligand model showed greater accuracy in predicting aGVHD than the ligand-ligand model and the difference was observed both in grade II-IV and grade III-IV severe aGVHD (II-IV: p=0.009;III-IV: p=0.012; **Table 3**). The number of donor aKIR was identified as a potent protective factor for aGVHD (II-IV: p=0.019, HR=0.156;III-IV: p=0.024, HR=0.165). Furthermore, the benefits increased with the increase in the total number of donor aKIR (**Table 4**). Dramatically, no aGVHD occurred in patients with receptor-ligand mismatch and the greatest total number of donor aKIRs, indicating a synergistic effect of KIR receptor-ligand mismatch and donor aKIRs, providing the strongest protective effect for aGVHD. Dummy **Figure 4**.

**Table 3:** Prediction of transplant outcome with killer cell immunoglobulin-like receptor (KIR) ligand-ligand mismatch (KLM) versus receptor-ligand mismatch (RLM).

Outcome	Mismatch model		*p* value comparing KLM vs RLM
	KLM	RLM	
Relapse ra/te	8.7%	6.6%	0.039
II-IV aGVHD	13.0%	9.8%	0.009
III-IV aGVHD	6.5%	4.9%	0.012
3yr-Overall survival	80.4%	78.7%	NS

**Table 4:** Effect of total number of donor aKIRs on transplant outcome.

Outcome	Number of donor aKIRs						*p* value
	1	2	3	4	5	5 aKIRs +KLM	
II-IV aGVHD	36.4%	25.0%	16.7%	16.6%	10.8%	0%	0.019
Relapse rate	26.3%	25.0%	16.7%	10.5%	10.0%	6.7%	0.037
3yr-Overall survival	54.5%	50.0%	66.7%	84.2%	85%	93.8%	NS

Among patients who survived more than 100 days after transplantation, 4 patients (5.1%) developed cGVHD. Univariate analysis and multivariate analysis did not find statistically significant factors associated with cGVHD.

### Relapse rate

After a median follow-up time of 2.0 years (range, 0.1–5.0 yr), 10 patients (12.7%) relapsed. Factors that may affect the incidence of RR were all taken into consideration as confounders in the multivariate analyses, including disease status (CR1 at transplantation or not), HLA-matching, donor type (related or unrelated), development of acute or chronic GVHD.

**Figure 5:**
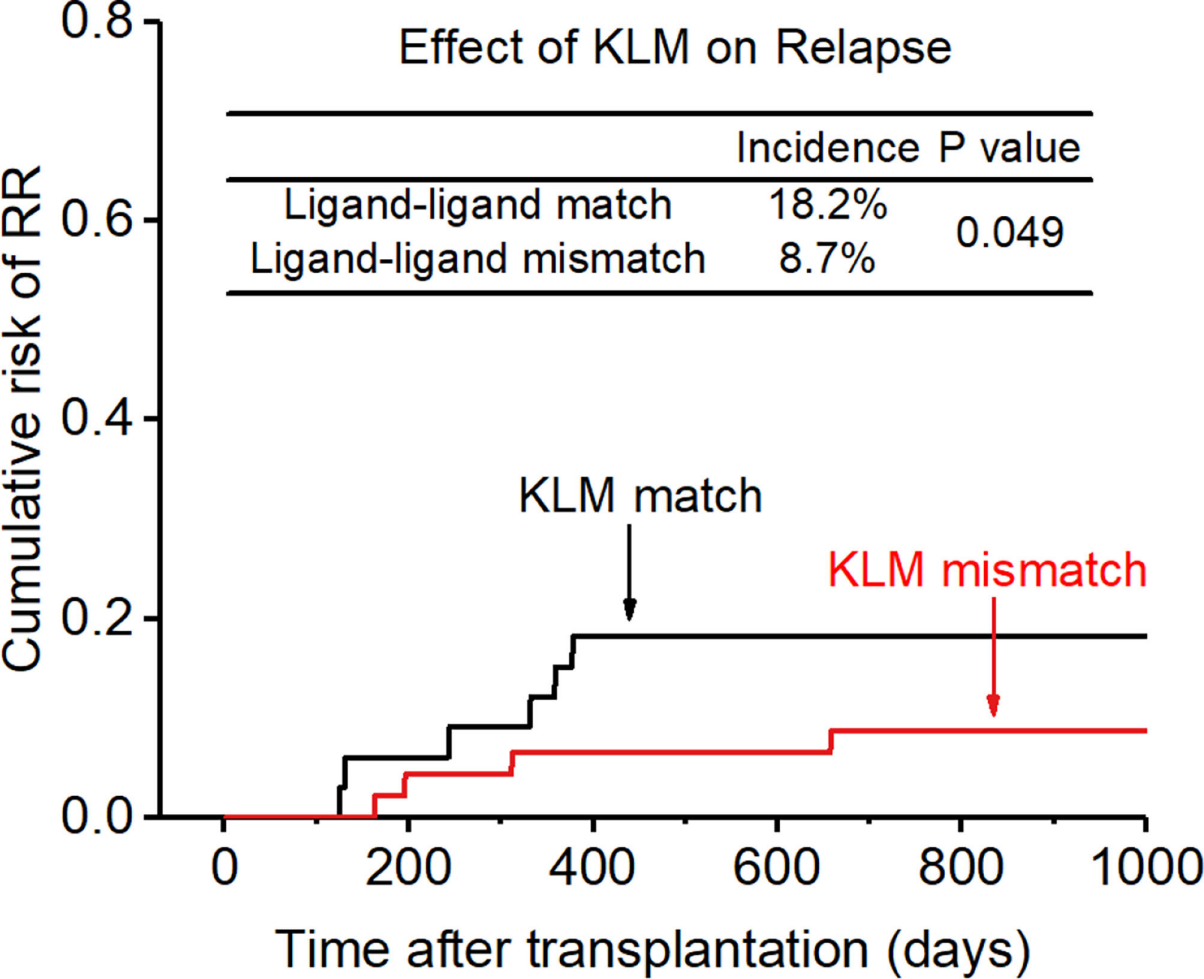
Effect of killer cell immunoglobulin-like receptor (KIR) ligand-ligand mismatch (KLM) on relapse rate (RR).

**Figure 6:**
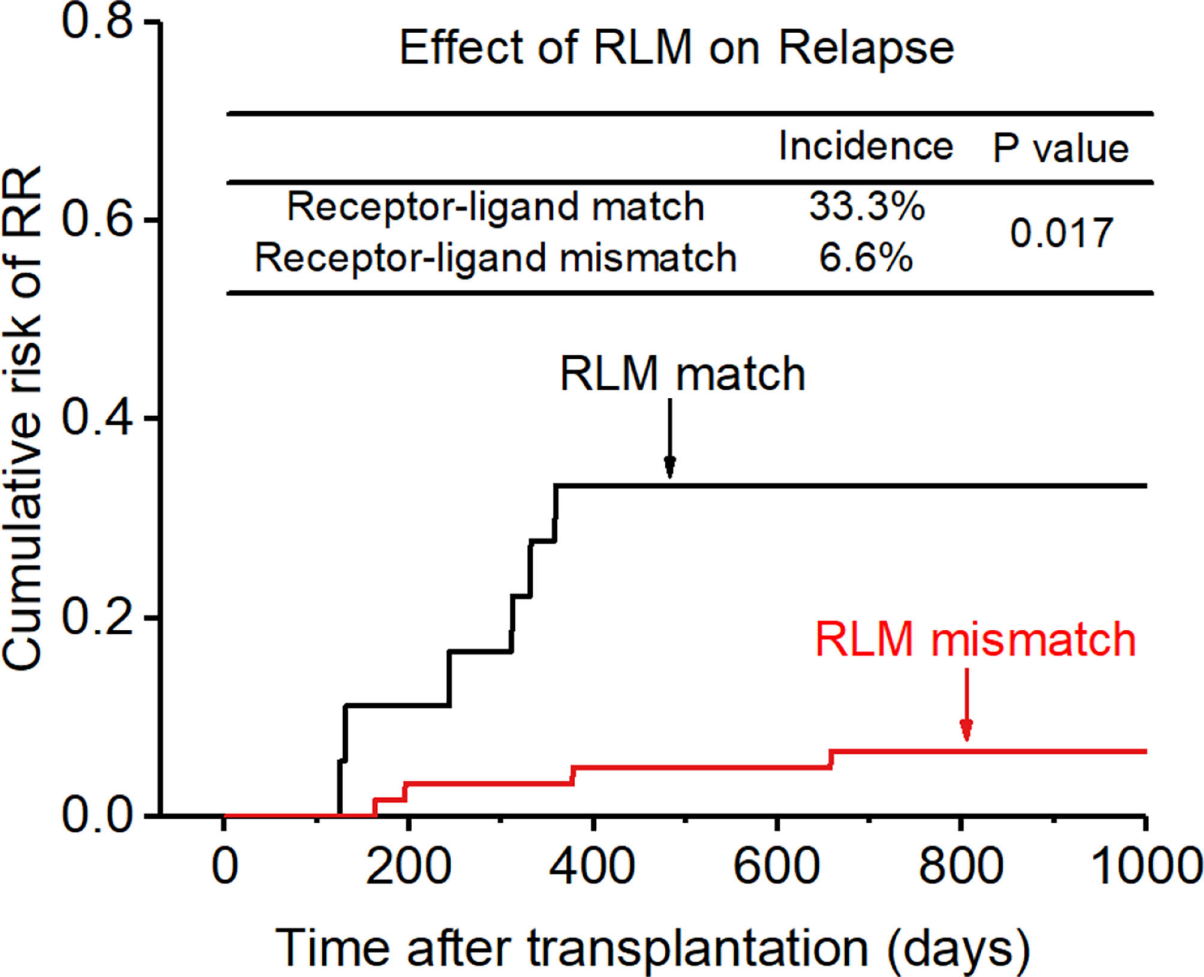
Effect of killer cell immunoglobulin-like receptor (KIR) receptor-ligand mismatch (RLM) on relapse rate (RR).

**Figure 7:**
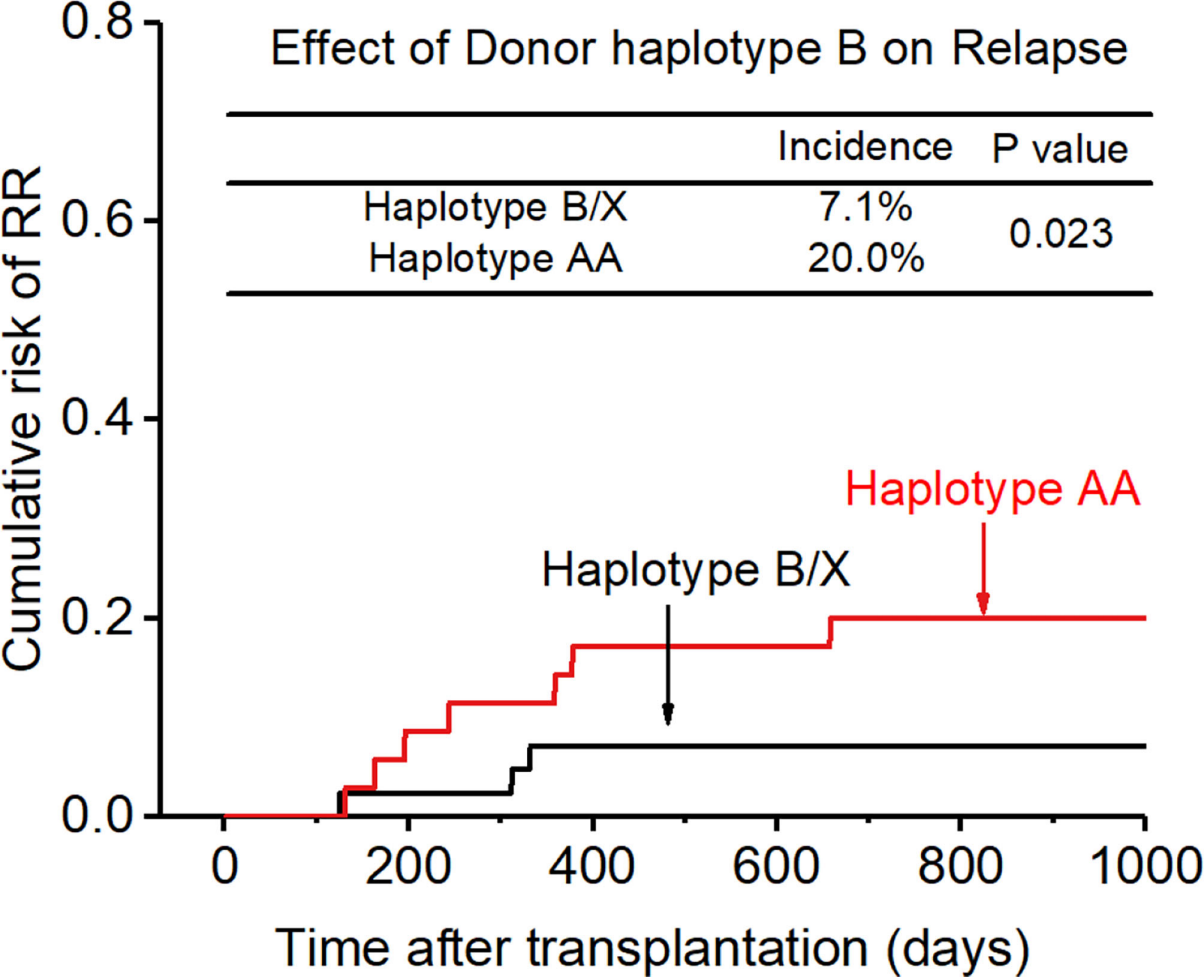
Effect of killer cell immunoglobulin-like receptor (KIR) haplotype-B on relapse rate (RR).

After adjusting for all these related factors, KIR ligand-ligand matching and receptor-ligand matching were independent risk factors for relapse for the entire cohort. (KLM: 8.7% vs 18.2%, HR:0.686, p=0.049, **Figure 5**; RLM: 6.6% vs 33.3%, HR:

0.200, p=0.017, **Figure 6**), and the accuracy of the relapse rate prediction was more evident in the RLM model (p = 0.039; **Table 3**). The number of donor aKIR and KIR haplotype B was associated with a lower incidence of relapse after adjusting for confounding factors (number of donor aKIR: HR:0.211, p=0.037, **Table 4**; haplotype-B: HR:0.174, p=0.023, **Figure 7**). Donor-recipient receptor-ligand mismatch combined with the greatest total number of donor aKIRs were shown to have the lowest relapse rate (6.67%; **Table 4**). In addition, patients with favorable/intermediate cytogenetic abnormalities experienced a lower relapse rate than patients with unfavorable cytogenetic abnormalities according to the multivariate analyses (HR:3.568, p=0.007). The CI for relapse was also lower in patients who received HSCT when at CR1 (HR:2.201, p=0.018). No significant differences in relapse rate were found with any of the other associated factors taken into consideration.

In addition, KIR ligand-ligand mismatch (CR1:6.9% vs 10.0%; >CR1:11.8% vs 30.8%), KIR receptor-ligand mismatch (CR1:5.3% vs 18.2%; >CR1:8.7% vs 57.1%), donor aKIR (CR1:5.9% vs 13.3%; > CR1:10.0% vs 40.0%), KIR haplotype B (CR1:3.8% vs 13.0%;>CR1:12.5% vs 28.6%) had a protective effect on recurrence after transplantation whether the patient disease status was at CR1 or not.

### Overall survival and NRM

**Figure 8:**
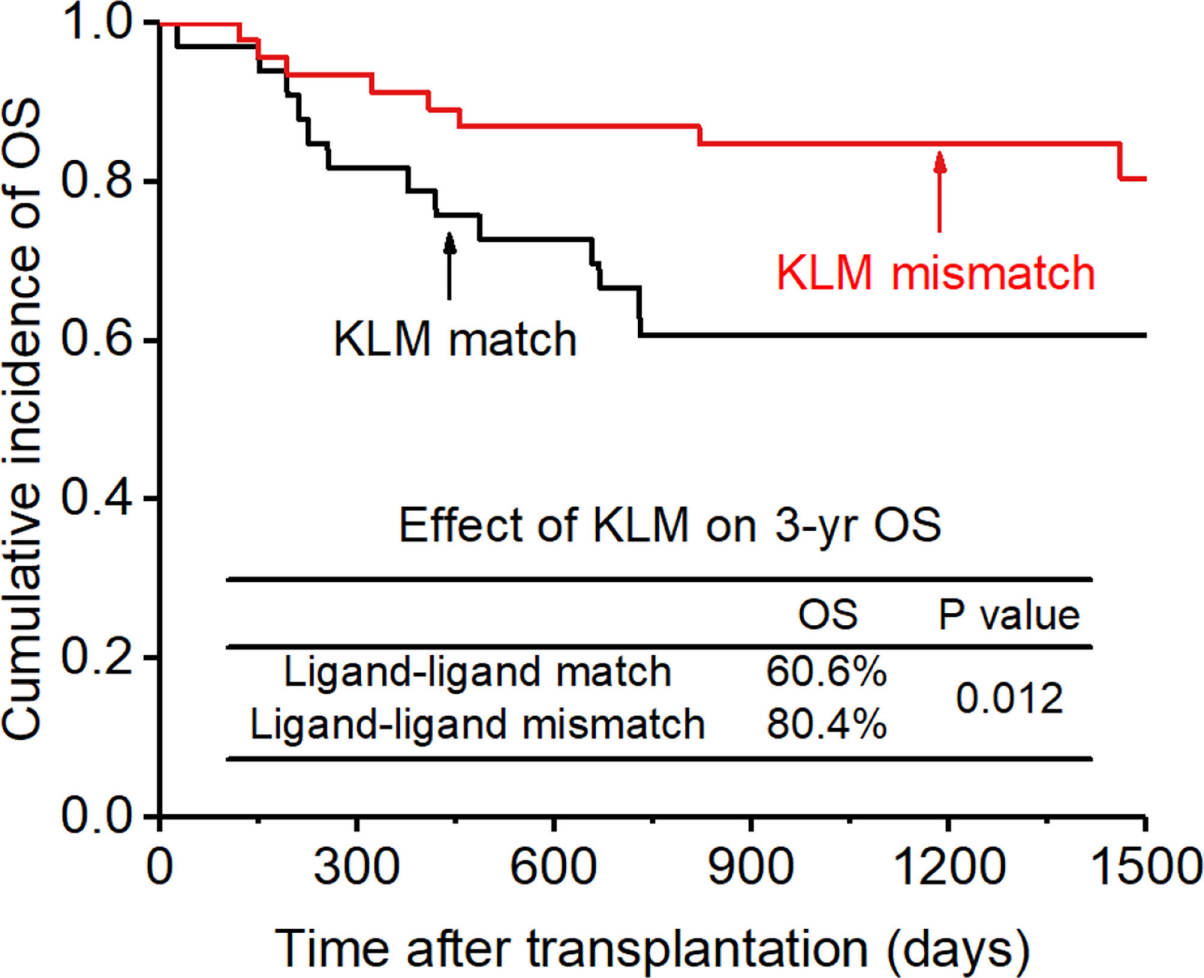
Effect of killer cell immunoglobulin-like receptor (KIR) ligand-ligand mismatch (KLM) on overall survival (OS).

**Figure 9:**
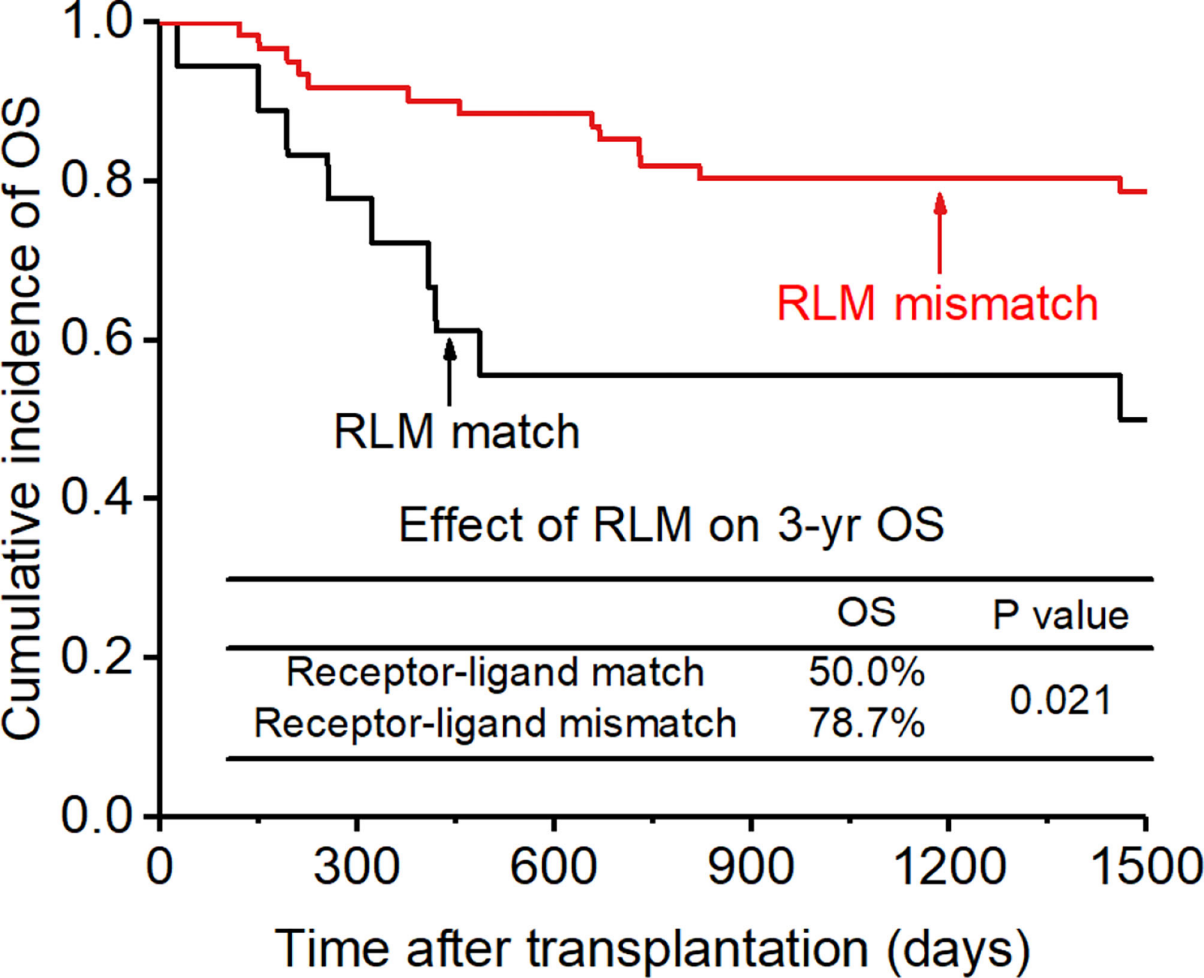
Effect of killer cell immunoglobulin-like receptor (KIR) receptor-ligand mismatch (RLM) on overall survival (OS).

**Figure 10:**
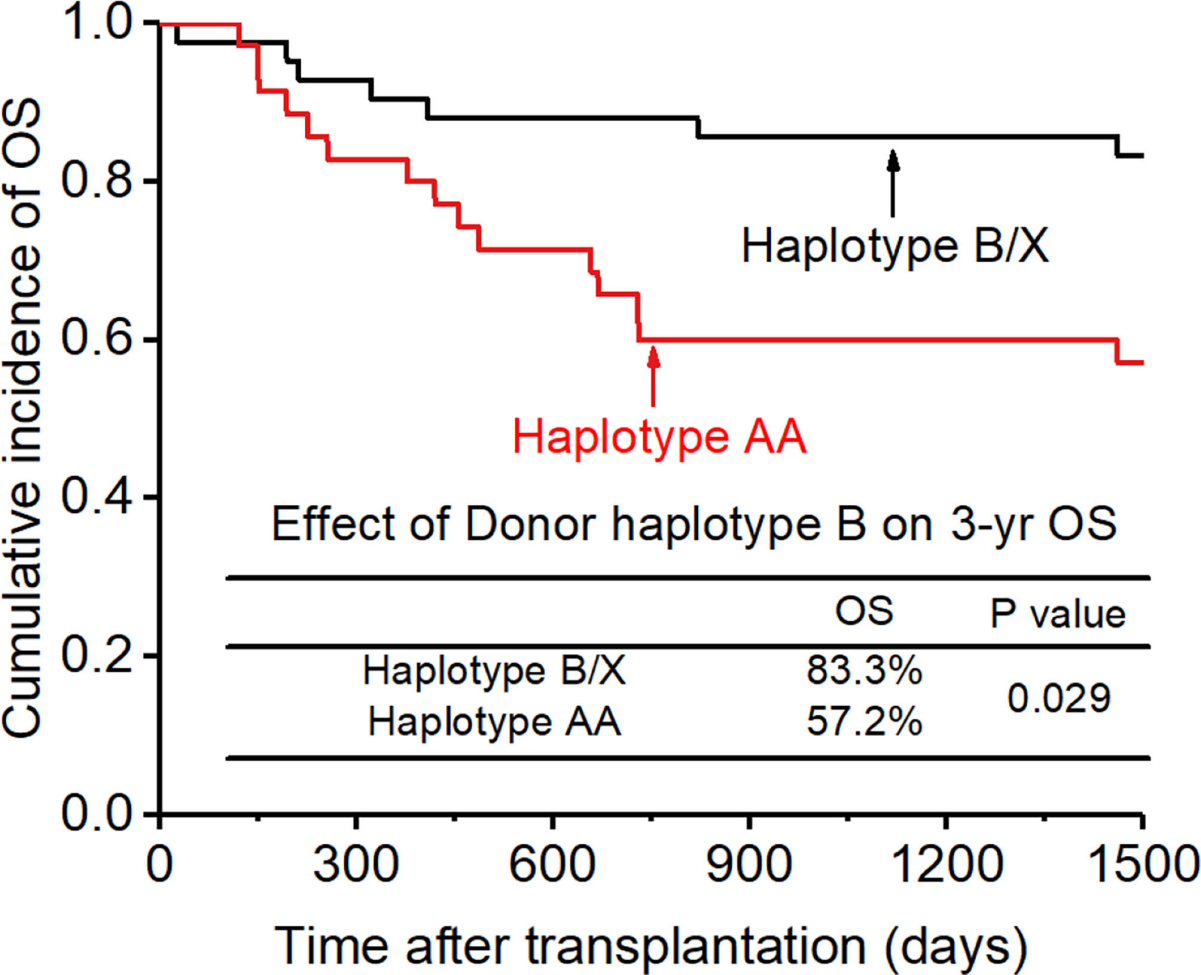
Effect of killer cell immunoglobulin-like receptor (KIR) haplotype-B on overall survival (OS).

The CI for 3-yr OS was 72.2% for all patients. Disease status (HR:2.654; p=0.007) and disease risk index (HR:4.132;p=0.006) were found to be the independent risk factors for the 3-yr OS in the multivariate analysis after adjusting with other factors. In addition, the 3-yr OS rate in patients with receptor-ligand mismatch or ligand-ligand mismatch donors was shown to be higher than those with the KIR ligand-ligand or recipient-ligand matching donors respectively (KLM: 80.4% vs 60.6%, HR:0.298, p=0.012, **Figure 8**; RLM: 78.7% vs 50.0%, HR:0.301 p=0.021, **Figure 9**). Donor haplotype-B was shown to be an independent protective factor for the 3-yr OS rate. (haplotype-B: p=0.029, HR:0.293, **Figure 10**). Although the number of donor aKIRs was not an independent risk factor for OS in the multivariate analysis because of the high mortality due to severe infections, patients with receptor-ligand mismatch combined with highest number of donor aKIRs still had the best 3-yr OS (1/16, 93.8%).

Among the 12 patients (15.2%) who experienced NRM, 2 (2.5%) died of severe aGVHD, 7 (8.9%) of severe infection, and 3 (3.8%) with primary poor graft function died from an intracranial hemorrhage. No variable was found to be a significant predictor of NRM.

## Discussion

This retrospective study addresses the impact of KIR-matching and KIR alleles on haplo-HSCT. The study reveals three major findings: (1) RLM and KLM may contribute to suppression of aGVHD and relapse, RLM offering best accuracy for prediction; (2) the number of donor aKIRs is a protective factor for aGVHD and disease relapse; (3) RLM associated with greater aKIR number can improve transplant outcome, especially regarding aGVHD and relapse.

According to differences in examination index, three hypotheses about KIR mismatching have been put forward and were taken into consideration in our study. The Receptor-Ligand Model (RLM) was proposed by Leung W et al., and is a competing model that aims to mismatch donor iKIR and recipient iKIR ligand to allo-reactivate donor NK cells (10, 23, 24). This model describes a KIR mismatch as the absence of a KIR ligand in the patient that is cognate by the donor’s KIR repertoire. The KIR Ligand-Ligand Model (KLM) is quite similar to the RLM, but defines a KIR mismatch as the absence of a KIR ligand in the recipient that is present within the donor ligand repertoire (a graft vs host [GvH] mismatch) (11, 25). There are few comparative analyses to assess the clinical relevance between these two relatively similar models (10). Our study indicates that both RLM and KLM can decrease the relapse rate and the incidence of aGVHD in AML patients after HSCT, which confirmed the effect of NK cell alloreactivity in leukemia elimination and GVHD protection (26, 27). Besides, RLM can more accurately predict relapse and aGVHD than KLM. There are several possible reasons for the difference. First, as KLM only tests HLA-I molecules, the model relays on the ligand to infer its corresponding ligand. Actually, there is disparity between the KIR repertoire and self-ligand repertoire, which means donor or recipient may either expressed an inhibitory KIR but have no corresponding ligand or expressed a KIR ligand but not the corresponding KIR. When donor NK cells do not express or have low expression of the corresponding KIR receptor according to HLA analysis, the prediction of the mismatch model may be hampered. Second, HLA-genotyping usually includes only HLA-C1 -C2, -BW4 and -A3/11 in clinical practice, leaving out the influence of HLA-G which is rare to be tested in clinical practice now. Besides the ligands for 2DL4 and 2DL5 or 3DL3 is still unknown, thereby reducing the precision of the mismatch prediction (10, 28–30). Some studies indicated that the effect of GVL induction in KLM applies to AML patients but not ALL patients, while the RLM antileukemia effect can be seen both in myeloid and lymphoid leukemia, thus supporting the greater advantage of RLM in predicting the risk of recurrence (6, 10, 11, 25). However, the same advantage from RLM was not observed for OS, which may be due to the higher mortality and severe GVHD in RLM patients than in KLM patients. The mortality from severe infections was slightly higher in RLM patients compared with KLM patients, but it did not reach statistical significance (severe infection: 11.5% vs 8.9%). The reason for the increased risk in severe infections may be the stronger cytotoxicity of allo-NK cells towards APCs predicted by RLM. Though APC killing may result in the suppression of aGVHD, it also hampers antigen presentation to T cells, which overwhelms the protective effect of activated NK cells on infection. The higher mortality from severe infections can also be seen in patients with greater total number of donor aKIRs, which can also be ascribed to the higher cytotoxicity of NK cells towards APCs, resulting in inefficiency at improving OS after HSCT. The small size of our patient cohort and the extremely low occurrence of death resulting from severe infection in our patients prevented to further study the difference in the effect of KLM and RLM in severe infection.

It is to be noted that the influence of donor-recipient KIR mismatching on GVHD remains extremely controversial and that opposite conclusions have been proposed by different studies (31). Our study found that KIR mismatching can significantly reduce GVHD occurrence after HSCT. It is worth emphasizing that all of our patients were given ATG or ATG-F for *in vivo* T cell depletion. Ruggeri et al. demonstrated that KIR-incompatibility could prevent the occurrence of GVHD after transplantation in patients or mice with T cells removed from the graft by *in vitro* CD34+ cell sorting, which highlights the importance of T cell depletion in NK cell alloreactivity towards GVHD (11, 25). Large number of donor-derived T cells may affect NK cell activity through cytokine selection and start the process of GVHD before the killing of recipient APCs by alloreactive NK cells occurs. Besides, KIR expression on NK cells may be higher when T cells are depleted in the graft (32), which may be due to the loss of cytokines competitors of NK cells after T lymphocyte removal (33), thus accelerating the maturation of NK cells and intensifying NK cell cytotoxicity towards T cells and APCs in the recipient, leading to GVHD suppression. Our study indicated the importance of large doses of ATG or ATG-F for *in vivo* T cell depletion by NK cell- induced lysis for removing host-type dendritic cells responsible for triggering GVHD and attacking residual host lymphohematopoietic cells, including T cells, which are responsible for graft rejection. Besides, neither ATG nor ATG-F had any influence on the effect of KIR-mismatch on aGVHD (**Supplementary Table 2**).

The RLM model only takes iKIRs into consideration, which overlooks the influence of aKIRs. Some studies have already confirmed the influence of some genotypes of aKIRs, such as 3DS1, 2DS1, 2DS3 etc., on HSCT outcome (14, 15, 34). Nevertheless, most studies mainly focused on the influence of iKIRs or aKIRs separately, preventing a better understanding of the role of aKIRs together with iKIRs in NK cell alloreactivity. Though NK cell function is predominantly modulated by iKIR mismatch, aKIRs can activate NK cells when encountering its ligand. Our study took aKIRs as the complement for the defect in only iKIR-based mismatch models and found that a greater number of donor aKIRs can result in lower relapse rate and lower incidence of aGVHD, and that this benefit can gradually increase in parallel with the increase in donor aKIR numbers. Association of RLM with greater donor aKIR number can lead to the best outcome, both by reducing RR and GVHD. Expression of more aKIRs on the donor NK cells increases the probability and intensity of activation of NK cells, thus increasing NK cell alloreactivity towards leukemia cells and GVHD induction cells, and leading to better transplant outcome compared to only taking into account the iKIR : HLA pairs. Patients with iKIR mismatch and more donor aKIRs can achieve the strongest allo-NK cell activation and the best HSCT prognosis. Our study suggested that the role of aKIRs cannot be neglected and should be taken into consideration in donor selection. However, some ligands for aKIRs remain unknown, which hampers further accurate definition and building of the scoring model for the new iKIR : HLA mismatch plus aKIR : HLA match model, thus, further studies are needed to better understand NK cell activation.

Heterogeneity of patients and donors hampers donor selection strategy based on only one indicator. Besides KIRs, related factors like donor sex, donor age, HLA-matching and some social factors like the intensity of the donor’s intention to donate, are inevitable and should be all taken into consideration of clinicians. Hence, our study laid the cornerstone for further studies about risk assessment model considering KIRs and other related factors and assessment of the priority over different influencing factors. Besides, our study highlight the importance of prospective studies on advanced interfere treatment to “high-risk” patients identified by KIR mismatch and KIR alleles, boosting the translation of our results from bench to bedside.

Our study has some limitations. This is a retrospective study coming from a single center, the population studied is homogeneous as all patients had AML, and the cohort is small. Thus, the results cannot be generalized to all allo-HSCT patients, and further studies in larger cohorts and in a more diverse population will be needed to fully understand the influence of KIR genotype variants on the prognosis of HSCT. Nevertheless, our study provides further support for a clinically applicable donor selection strategy to improving allo-HSCT outcome in patients with AML, especially for ATG or ATG-F based haplo-HSCT. Therefore, we can recommend selecting a donor with both RLM and the most aKIRs to reach the strongest alloreactivity of NK cells and promote better transplantation outcome.

## Data availability statement

The raw data supporting the conclusions of this article will be made available by the authors, without undue reservation.

## Ethics statement

The studies involving human participants were reviewed and approved by Human Investigation Committee of the Fujian Medical University Union Hospital. Written informed consent to participate in this study was provided by the participants’ legal guardian/next of kin.

## Author contributions

All authors contributed to the study conception and design. Material preparation, data collection and analysis were performed by YZ, CY and HZ. The first draft of the manuscript was written by YZ and all authors commented on previous versions of the manuscript. All authors read and approved the final manuscript.
