# Supplementary material for: Association of iKIR-mismatch model and donor aKIRs with better outcome in haploidentical hematopoietic stem cell transplantation for acute myeloid leukemia
Source: Front Immunol. 2023 Jan 24;13:1091188. doi: 10.3389/fimmu.2022.1091188 (PMC9904326; doi:10.3389/fimmu.2022.1091188)
Supplement: Supplementary file 1 [file DataSheet_1.pdf]

Supplementary Material

Supplementary Table1. Three types of hypothesis for KIR mismatch

Mismatch model	Proposer	Relationship	Test ordered	Definition of mismatch
**Ligand mismatch**	Ruggeri in 2002[1]	Donor KIRL vs. recipient KIRL	Donor HLA recipient HLA	Miss KIRL in a recipient for a KIRL that is present in donor
**Receptor mismatch**	Gagne in 2002[2]	Donor KIR vs. recipient KIR	Donor KIR recipient KIR	Missing KIR in a recipient for a KIR that is present in donor
	McQueen in 2007[3]	Donor KIR haplotype vs. recipient KIR haplotype	Donor KIR haplotype recipient KIR haplotype	A mismatch between donor and recipient for the B KIR haplotypes
**Receptor-ligand mismatch**	Leung in 2004[4]	Donor KIR vs. recipient KIRL	Donor KIR recipient HLA	Missing KIRL in a recipient for a KIR that is present in donor

Supplementary Table2. Influence of ATG or ATG-f on the effect of KLM or RLM on aGVHD

Mismatch model	II-IV aGVHD		P value	95% (CI)
	ATG	ATG-f		
**Ligand-ligand mismatch**	11.4%	13.0%	0.190	1.785 (0.765-4.776)
**Receptor-ligand mismatch**	10.2%	9.5%	0.400	1.231 (0.522-3.011)

Supplementary Figure1. Three KIR/KIR ligand models defined to predict potential KIR based NK cell alloreactivity. The HLA class I typing of donor/recipient pairs permits the identification of KIR ligand mismatches in the graft-versus-host (GvH) direction in HLA-mismatched HSCT, as defined by the KIR ligand-ligand model or by the KIR ligands in recipients that show missing KIR ligands (A). KIR genotyping of donors and recipients permits the assignment of donor and/or recipient KIR genes that impact the HSCT outcome, as defined by the KIR gene-gene model or KIR receptor-receptor model (B). Genotyping of HLA class I genes in donor/recipient pairs and of KIR genes in donors permits the identification of KIR receptor-ligand model (C).

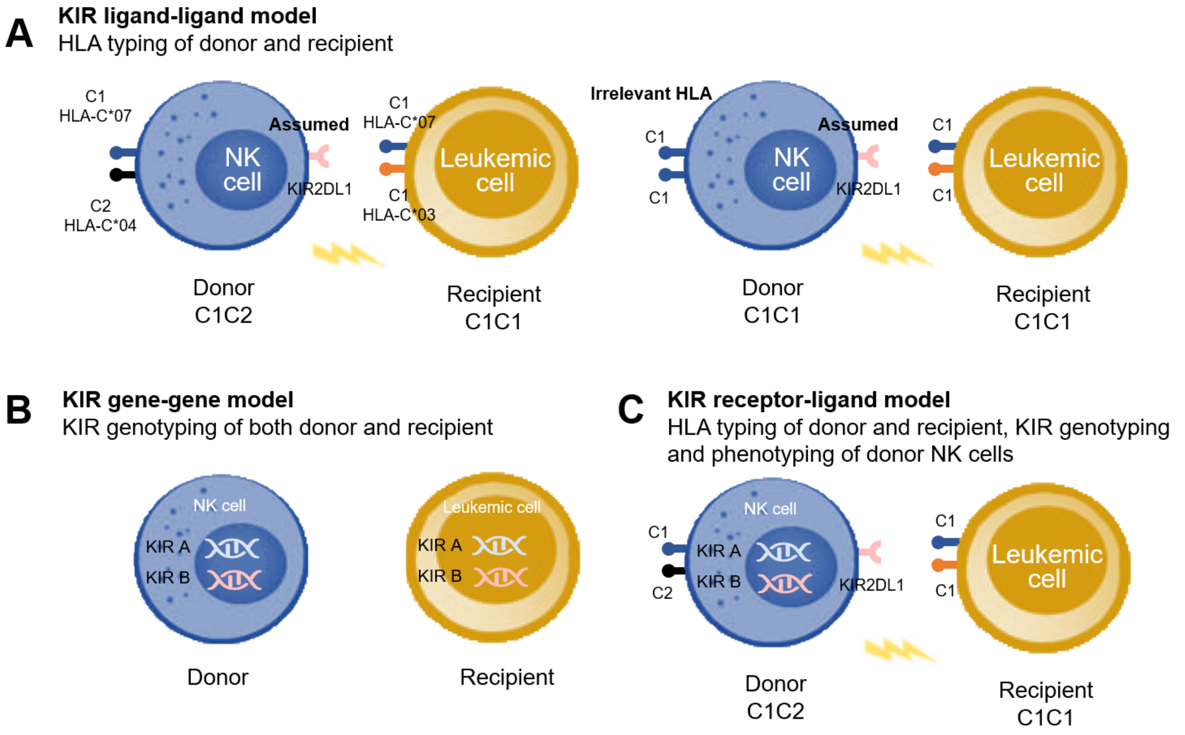

References

[1] L. Ruggeri, M. Capanni, E. Urbani, K. Perruccio, W.D. Shlomchik, A. Tosti, S. Posati, D. Rogaia, F. Frassoni, F. Aversa, M.F. Martelli, and A. Velardi, Effectiveness of donor natural killer cell alloreactivity in mismatched hematopoietic transplants. Science 295 (2002) 2097-2100.

[2] K. Gagne, G. Brizard, B. Gueglio, N. Milpied, P. Herry, F. Bonneville, M.L. Cheneau, N. Schleinitz, A. Cesbron, G. Follea, J.L. Harrousseau, and J.D. Bignon, Relevance of KIR gene polymorphisms in bone marrow transplantation outcome. Human Immunology 63 (2002) 271-280.

[3] K.L. McQueen, K.M. Dorighi, L.A. Guethlein, R. Wong, B. Sanianwala, and P. Parham, Donor-recipient combinations of group A and BKIR haplotypes and HLA class I ligand affect the outcome of HLA-matched, sibling donor hematopoietic cell transplantation. Human Immunology 68 (2007) 309-323.

[4] W. Leung, R. Iyengar, V. Turner, P. Lang, P. Bader, P. Conn, D. Niethammer, and R. Handgretinger, Determinants of antileukemia effects of allogeneic NK cells. Journal of Immunology 172 (2004) 644-650.
